# Cluster randomised controlled trial of ‘whole school’ child maltreatment prevention programme in primary schools in Northern Ireland: study protocol for *Keeping Safe*

**DOI:** 10.1186/s12889-018-5492-8

**Published:** 2018-05-03

**Authors:** Aisling McElearney, Aoibheann Brennan-Wilson, Christina Murphy, Phyllis Stephenson, Brendan Bunting

**Affiliations:** 1 National Society for the Prevention of Cruelty to Children, Northern Ireland Regional Office, Lanyon Building, North Derby Street, Belfast, BT15 3HN Northern Ireland; 2 Bamford Centre, Psychology Research Institute, Ulster University, Coleraine, Northern Ireland

**Keywords:** Child maltreatment, Prevention, Whole school education programme, Cluster randomised controlled trial, School-based programme, Protocol, United Kingdom

## Abstract

**Background:**

Child maltreatment has a pervasive, detrimental impact on children’s wellbeing. Despite a growing focus on prevention through school based education, few programmes adopt a whole- school approach, are multi-component, seek to address all forms of maltreatment, or indeed have been robustly evaluated. This paper describes a cluster randomised controlled trial designed to evaluate a school based child maltreatment prevention programme: ‘Keeping Safe’ in primary schools in Northern Ireland. The intervention has been designed by a non-profit agency. Programme resources include 63 lessons taught incrementally to children between four and 11 years old, and is premised on three core themes: healthy relationships, my body, and being safe. There are programme resources to engage parents and to build the capacity and skills of school staff.

**Methods/design:**

A cluster Randomised Controlled Trial (RCT) will be conducted with children in 80 schools over a two-year period. The unit of randomisation is the school. Schools will be allocated to intervention or wait-list control groups using a computer-generated list. Data will be collected at three time points: baseline, end of year one, and end of year two of programme implementation. Primary outcomes will include: children’s understanding of key programme concepts, self-efficacy to keep safe in situations of maltreatment, anxiety arising from programme participation, and disclosure of maltreatment. Secondary outcomes include teachers’ comfort and confidence in teaching the programme and parents’ confidence in talking to their children about programme concepts.

**Discussion:**

This RCT will address gaps in current practice and evidence regarding school based child maltreatment prevention programmes. This includes the use of a whole- school approach and multi-component programme that addresses all maltreatment concepts, a two-year period of programme implementation, and the tracking of outcomes for children, parents, and teachers. Methodologically, it will extend our understanding and learning in: capturing sensitive outcome data from young children, adapting and using standardised measures with children of different ages, the use of school level administrative data on staff reports/children’s disclosure of maltreatment as behavioural outcomes, and the conduct of complex trials within the busy school environment.

**Trial registration:**

ClinicalTrials.gov: NCT02961010 (Retrospectively registered 8 November 2016).

## Background

Child maltreatment remains a significant global problem [1, 2]. Prevalence estimates indicate that 12.7% of children experience sexual abuse, 22.6% physical abuse, 36.3% emotional abuse and 16.3% physical neglect [3]. While this data concerns over one billion children aged between two and 17 years worldwide, these self-report estimates are considered to under-represent children’s experience; up to 30 times in the case of sexual abuse and 75 times in cases of physical abuse [4, 5]. Children with disabilities are at greater risk [6]. Thirty-five per cent of children experience bullying while 15% report experiencing cyber-bullying [7], which is also considered to be underreported [8]. Moreover, many children experience multiple types of maltreatment across their childhood, perpetrated by different people in different settings and contexts [9]. Trend data indicates increased reporting of emotional abuse and neglect, as well as sexual abuse carried out online or using digital technology, and abuse perpetrated by other children [10–14].

Maltreatment experiences have been shown to have pervasive detrimental impacts on health and wellbeing outcomes in the short and longer term. This includes relationship, reproductive and sexual behaviour disorders [15], suicide, bi-polar disorders, and alcohol and drug abuse [16]. Children who experience physical abuse, sexual abuse or neglect are more likely to develop depression and anxiety in adulthood [17] while experiencing emotional maltreatment in childhood is significantly associated with developing severe, early onset chronic depression in adulthood [18]. Children living with domestic violence are also at greater risk of experiencing these other forms of maltreatment [19]. Furthermore, experiencing school bullying and cyberbullying in childhood is associated with adult drug use [20], stress and suicide ideation [14]. Such experiences and their impact have been shown to have significant financial costs to the individual as well as society, in terms of healthcare, social care, education, criminal justice and lost productivity [21–23]. The most recent research reported in the United Kingdom estimates the average lifetime incidence cost of non-fatal child maltreatment by a primary caregiver at £89,390 [24].

### Prevention – School based programmes

Acknowledgement of the global prevalence, impact and costs of child maltreatment has led to a greater focus on prevention, and indeed school based prevention within the context of a public health framework, which seeks to address risk factors at the level of community and society as well as the individual child [25–28]. Despite the development of such school based programmes in high income countries over the past 40 years, research confirms significant gaps in practice and evidence [28–32].

Significantly, the majority of these programmes published in the research literature have been developed in the United States, Canada, Australia and New Zealand [29, 32]. The majority are classroom or curricular based as distinct from ones that focus on the whole school/organisation, are of relatively low dose [29, 30], focus on one type of maltreatment and are considered to lack coherence and breadth [12, 28, 33]. Moreover, these programmes are more likely focussed on building protective knowledge, awareness and skills of older children/young adults. It is reported that less than 10 % of inter-personal/relationship violence prevention programmes target elementary or middle school children [14, 33].

Overall, evidence indicates that school based prevention education programmes concerned with child sexual abuse [29], domestic abuse [30] and relationship and dating violence [32, 33] can lead to significant improvements in protective knowledge and attitudes in the short term. Significant behavioural outcomes were reported only in a small minority of programme evaluations concerned with bullying perpetration and victimisation [31, 34], perpetration of sexual violence [33] and disclosures of sexual abuse [29]. Methodological shortcomings in the evaluation of programmes have led to a lack of evidence as to whether school based prevention programmes actually reduce maltreatment [35]. More rigorous evaluation is required to address this question of effectiveness [13, 29, 30, 33, 36, 37] and should include the use of experimental designs [29, 35], appropriate standardised validated measures [14, 26, 30–32], measurement of behavioural outcomes [28, 29, 32, 33, 35], exploration of negative/adverse effects and should include follow up in the longer term [29, 32].

**Fig. 1:**
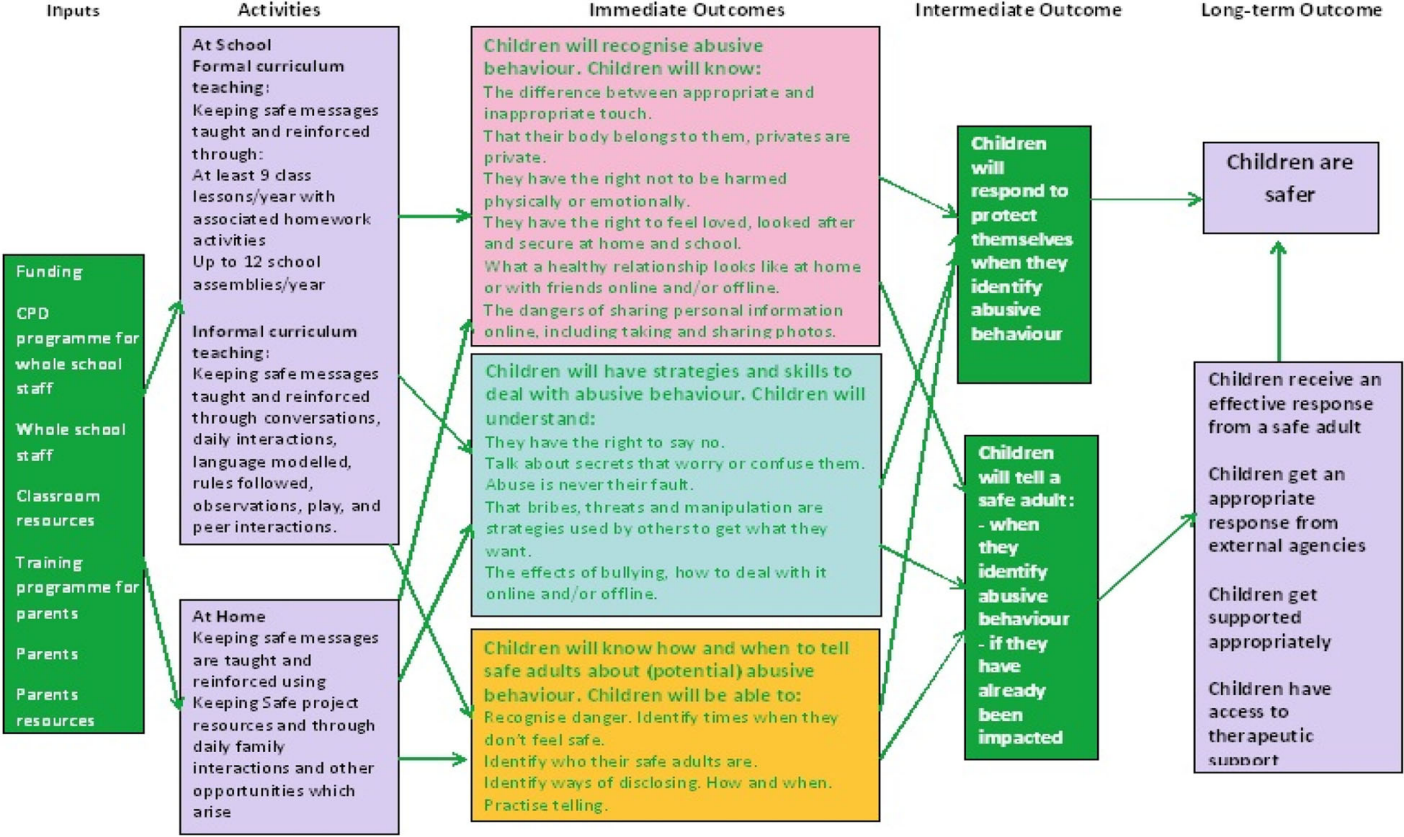
Logic Model: Keeping Safe programme for children 4–11 years

This paper concerns the evaluation of a comprehensive multi-component whole school maltreatment prevention programme ‘Keeping Safe’, developed in Northern Ireland. Programme development, led by a non-government agency, engaged local experts and key stakeholders in the development of a systematic logic model (see Fig. 1) informed by the principles associated with effective prevention interventions [38, 39], and a review of international research evidence and practice [12, 28, 29, 31–34, 40, 41], which sought to identify effective components of school based maltreatment prevention programmes and gaps in current practice. Moreover, local evidence captured through a comprehensive needs assessment with stakeholders in Northern Ireland [42–46] was also used to inform the development of a programme model that is culturally appropriate and attuned to the child protection practice environment that exists in Northern Ireland [25, 26, 30].

### Aim

The aim of this study is to conduct a randomised controlled trial to determine the effectiveness of the *Keeping Safe* programme.

### Objectives

#### Primary objectives

To determine if exposure to the *Keeping Safe* programme impacts:

- children’s self-reported knowledge and understanding of the following keeping safe concepts: bullying, neglect, physical, emotional, domestic and sexual abuse
- children’s self-reported self-efficacy to keep safe in situations of bullying, neglect, physical, emotional, domestic and sexual abuse
- children’s self-reported skills as measured by the proxy skills measure ‘What If Situations Test’ to keep safe in situations of abuse/maltreatment
- children’s disclosure and telling/ staff observation as measured by school level administrative safeguarding data including concerns noted, disclosures made, referrals
- children’s self- reported anxiety

#### Key secondary objectives

To determine if engagement with the Keeping Safe programme impacts:

- teachers’ self-reported knowledge in teaching keeping safe messages
- teachers’ self-reported confidence and comfort in their own skills to manage sensitive issues in relation to Keeping Safe in the classroom
- parents’ self-reported knowledge of and confidence to communicate with their child about keeping safe messages
- parents’ self-reported current and future confidence to communicate with their child about keeping safe messages

#### Further secondary objectives

To determine:

- if changes in children’s knowledge and understanding vary with reported whole school level programme implementation, and classroom level programme implementation
- how school context factors such as size, sector and management type, and teaching context factors; reported teacher access/use of continuing professional development training and resources and reported teacher knowledge, comfort and confidence in teaching keeping safe messages impact on children’s knowledge, understanding and skills, disclosures/referrals
- how parent characteristics; personal (gender) parenting profile (age of children, no of children) and home context (postcode) factors impact on children’s knowledge, understanding and skills, disclosures/ referrals
- how school context factors such as size, sector and management type, and professional experience factors (length of teaching service, nature of role in school) impact on teacher knowledge in teaching keeping safe messages, and confidence and comfort in their own skills to manage sensitive issues in relation to Keeping Safe in the classroom
- if access to, and use of the continuing professional development training and resources specifically designed for the Keeping Safe programme impact on teachers’ knowledge in teaching keeping safe messages and their confidence and comfort in their own skills to manage sensitive issues in relation to Keeping Safe in the classroom
- if implementing the Keeping Safe programme leads to change in school ethos/climate, and if this differs across schools
- how personal (gender) parenting profile (age of children, no of children, relationship to child) and home context (postcode) factors impact on parents’ knowledge of and confidence to communicate with their child about keeping safe messages

## Trial design

This study has been approved by the NSPCC Research Ethics Committee R/15/67and is funded by the Department of Education in Northern Ireland. The Keeping Safe trial is designed as a two-arm cluster randomised control trial using a wait-list control group with a 1:1 allocation. The unit of analysis will be the school but analysis will also be conducted at the level of the individual, while taking into account the nesting effects of class and school.

### Study setting

This trial seeks to provide the evidence to inform Department of Education Northern Ireland policy regarding the development of prevention education across the Northern Ireland school system. In order to ensure a representative sample and address religious segregation/equality issues specific to Northern Ireland, schools will be recruited from across the five regions of the Education Authority in Northern Ireland and will take account of the four main School Management Types (Roman Catholic Maintained, Controlled, Grant Maintained Integrated, and Irish-Medium).

### Eligibility criteria

#### Inclusion criteria

- Children aged four to 11 years old attending mainstream sector primary school in Northern Ireland whose school has accepted the invitation to take part in the RCT and whose parents have provided consent for their participation.

#### Exclusion criteria

- Children attending special sector primary schools in Northern Ireland.
- Children aged four to 11 years old attending mainstream sector primary school in Northern Ireland whose school has not been invited/ rejected the invitation to take part in the study
- Children aged four to 11 years old attending mainstream sector primary school in Northern Ireland whose school has accepted the invitation to take part in the study and whose parents, or they personally, have not provided consent for participation.

### Randomisation

Randomisation will be carried out by an independent consultant and will be conducted using a computer-generated list of numbers. The allocation will be emailed to the Principal Investigator who will then email individual notifications to schools within a designated timeslot on 1 day. Entire schools will be randomised to the intervention or wait-list control following baseline data collection with stratification by School Management Type and school enrolment size with a 1:1 allocation. This will be achieved as follows:

- The schools will be sorted by management type and, within management type, by enrolment numbers
- Schools will be assigned to pairs starting from the top of the sorted list. For example, the two largest catholic maintained schools will be assigned to ‘Pair 1’; the third and fourth largest to ‘Pair 2’ etc.
- A random number between 0 and 1 will be assigned to each school (the random number being generated within SPSS)
- Within each of the pairs the school with the smallest random number will be assigned to the intervention group and the other school from the pair will be assigned to the wait-list control group

### Intervention: Keeping safe

*Keeping Safe* is a comprehensive multi component ‘Whole-School’ programme designed to teach children aged four to 11 years old how to keep safe from all forms of maltreatment, including: neglect, sexual abuse carried out online or using digital technology, abuse perpetrated by other children, and bullying. This is premised on identified priority gaps in existing programmes [12, 13, 22, 26, 33, 37] and the fact that key messages have applicability across multiple forms of maltreatment [28, 47]. Table 1 outlines the key themes and messages taught to children and tailored to their age and developmental needs. For example in relation to sexual abuse, children are taught how to recognise unsafe situations and grooming behaviours such as the use of bribes and threats. Keeping Safe also teaches children that people they know and love can perpetrate abuse which is important as the majority of children will be abused by people they know rather than strangers [13]. Moreover, while the programme employs a range of methods to teach knowledge and understanding (for example books, role play and animations), significant time and content is devoted to skill development and practice; a key component of effective prevention programmes [22, 28, 41, 48].

**Table 1:** Overview of the Keeping Safe Programme: Themes and examples of key messages by age group

Theme description and Term	Examples of key messages for children by age-group
Healthy Relationships in Term 1: teaches children to recognise what a healthy relationship looks like (online or face to face), between adults at home, peers or friends. This theme addresses bullying behaviour and most forms of abuse.	4-6 yrs. the names of feelings, their hands are not for hurting, that no one has the right to hurt them, what to do if someone hurts them, what to do if they are worried about someone else being hurt.
	6-8 yrs. they have the right to be in a happy and caring environment, what a good friendship should look like, that it’s okay to say no to a friend, what to do if they feel hurt by anyone (even if it is an adult).
	8-11 yrs. the importance of having respect for others, the different types of bullying behaviour, the reasons for it and strategies for dealing with bullying, what cruelty is and how we can stop it, the problems that can occur with online friendships, how to recognise an unhealthy relationship, what domestic abuse is and know that it is wrong.
My Body in Term 2: focuses on sexual abuse. It teaches children to recognise when they don’t feel safe by explaining the difference between appropriate and inappropriate touch, the difference between secrets and surprises and how to identify if they are being tricked into doing something unsafe.	4-6 yrs. what private means and know the proper names for body parts, that we don’t share private parts, the difference between appropriate and inappropriate touch, they have the right to say no if their body gets a feeling that they don’t like, the difference in secrets and surprises.
	6-8 yrs. that privates are not for sharing, that their body belongs to them, to talk about secrets that upset them even if it involves someone they or their family know very well, no one should make them do things that they don’t want to, they will know how to say no, to recognise bribes and threats and know what to do.
	8-11 yrs. that their body belongs to them and they are entitled to privacy, that private areas should never be shared, the potential dangers with sharing photos online, to be able to identify the four main forms of abuse (neglect, sexual, physical and emotional), the problems some people face in telling about abuse.
Being Safe in Term 3: encourages children to talk about their feelings. They are taught how to seek help from a trusted adult when they don’t feel safe or realise what has happened to them is wrong or abusive. Children are also taught how and when to talk to safe adults and to keep telling until they get help.	4-6 yrs. what safe means and to explain the feelings they get when they are safe, identify situations that are safe and not safe, to tell someone if they are not feeling safe, that they should talk about worries, identify their safe adults, the importance of asking for help from an adult while online.
	6-8 yrs. recognise different body signs when they feel unsafe, how to keep themselves safe online, the importance of staying with a safe adult in public places, safe people to ask for help from in different situations, , not to make judgements based on appearances alone.
	8-11yrs. the difference in needs and wants, they have a right to feel safe and secure, how their body reacts when they are angry or feel threatened, what is safe to share online and offline, to tell an adult if they are being asked for personal information online or being asked to meet someone they have met online, what is meant by cyber bullying, how and why it happens and how to deal with it, to educate younger children in the school about ‘Keeping safe’.

The ‘Whole- School’ model adopted by Keeping Safe is increasingly advocated in policy documents [1, 2, 49] yet is relatively untested and lacks evidence of effectiveness [30]. This model supports the development of Keeping Safe as a multi-component programme with key components targeted at children and their parents as well as teachers and other whole school staff.

Teaching and learning resources seek to build the capacity of school staff to teach and reinforce messages through the formal and informal curriculum. These were designed to integrate into the existing ‘Personal Development & Mutual Understanding’ statutory component of the Northern Ireland Curriculum in primary schools and to the wider life of the school [30, 34]. The classroom based materials developed across three themes of healthy relationships, my body and being safe, include a wide range of materials including 63 lesson plans (nine for each class group per year) designed to teach key messages incrementally as the children progress through the 7 years of their primary school education from Primary one to Primary seven. This approach of providing curriculum support materials that are tailored to the age and developmental abilities of the children and build on prior learning is associated with effective prevention programming [29, 30, 48]. In practice, school leaders will deliver a prepared assembly, one of twelve available, to introduce the theme for the term. Each teacher will then deliver three age appropriate lessons to their class and ask the children to complete the accompanying homework with their parents or carers. The structured programme provides teachers with sufficient time and multiple opportunities to revisit and reinforce key messages; important elements of effective programmes [48].

Parental involvement, also associated with effective programmes [47, 50–53] is an integral component of Keeping Safe. Parents are engaged in directed homework activities with their children and are encouraged to attend a structured information session and expert workshops seeking to build their knowledge, skills and confidence to reinforce their children’s learning at school. Training and support for teachers and whole school staff, also an important component of the Keeping Safe programme [26, 30, 54], involves a blended package of training aimed at building the capacity of school leaders, teaching and non-teaching staff to teach and embed the programme in all aspects of school life.

### Wait list control group

The wait-list control group will continue to receive the Northern Ireland Personal Development & Mutual Understanding curriculum as usual for the duration of the trial. They will be advised to continue and record delivery of other related programmes/ resources already embedded in school practice, and asked not to take on any new programmes of this nature for the duration of the trial. They will participate in three rounds of data collection and will receive the programme once the evaluation ends.

### Recruitment and participants

The trial will adopt a random stratified sampling approach to school recruitment. The sample will be stratified according to the four main School Management Types (Roman Catholic Maintained, Controlled, Grant Maintained Integrated, and Irish-Medium) and the five regions of the Education Authority in Northern Ireland.

**Fig. 2:**
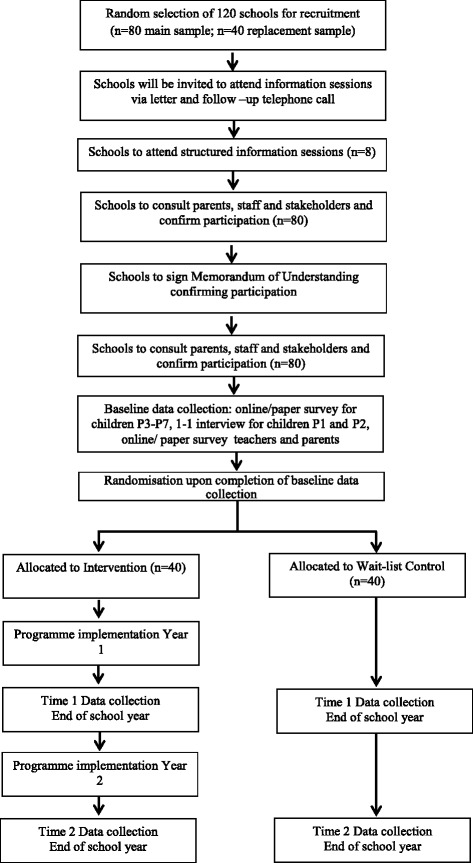
Flow of participants in Keeping Safe RCT

A total random sample of 120 primary schools will be identified for recruitment purposes (see Fig. 2). This will include a main sample of 80 schools and a replacement sample of 40 schools. If a school from the main sample declines their invitation, a matched replacement school will be invited to attend in their place. Additional samples will be identified as required until recruitment targets are met. It is envisaged that approximately 150 children will complete questionnaires per school at each data collection timepoint. The average enrolment number is 200 children per school but only a minority of children in the youngest primary year groups (Primary One and Primary Two) will take part in the study. Further to this, it is also anticipated that there will be absences among older year groups.

A member of the school leadership team will be invited to attend an information session about the programme. These sessions will include presentations from the project team on the programme content and delivery, and the RCT evaluation. School leaders will be provided with school planning resources including school development plan inserts, school calendars outlining key dates for programme implementation and evaluation as well as sample surveys/ programme resources to assist their informed decision-making regarding participation. To maximise recruitment, the project team will also follow up with phone calls and provide additional information and support/meetings as and if required. School leaders will be asked to consult their stakeholders and provide consent for their school to participate in the trial. They will then be asked to sign a Memorandum of Understanding that formalises expectations and responsibilities of schools and the project team for the conduct of the trial. Schools will then be asked to distribute evaluation information packs and consent forms to the parents of every child in their school.

### Sample size

The sample size calculation was based on an assumed intra-class correlation coefficient of 0.2, as observed in similar school-based preventative education programmes [29]. The calculation was also based on the primary outcomes of the study, i.e. changes in children’s knowledge, understanding, self-efficacy and proxy skills to keep safe from all forms of abuse. Other studies concerned with preventing sexual abuse that have used a cluster randomised design have reported an average increase of 0.63 standard deviations (ICC = 0.2) for knowledge of sexual abuse concepts, and an average increase of 0.60 standard deviations (ICC = 0.2) for proximal measures of safety skills (i.e. the What If Situations Test) in the intervention group. Programmes with a focus on domestic abuse prevention have reported increases of 0.3–1.3 standard deviations for knowledge of domestic abuse concepts in the intervention group [30]. These studies have also reported an odds ratio of 2.95 in relation to disclosures of abuse [29] while others have reported an odds ratio of 1.10 in relation to reductions in bullying victimisation and perpetration [40].

Power calculations for the current study indicate that approximately 40 schools are required in each arm of the study to detect a minimum detectable effect size of 0.19 standard deviation with 80% power and 95% significance. This will also allow for a minimum detectable percentage point difference of 2.7 for an outcome that is rare in the control group, at around 5% (i.e. disclosure of abuse).

There are several assumptions behind the calculations:

1. The average number of completed surveys per school will be around 150 per timepoint. (The average enrolment number is 200 per school, but the very youngest year groups will not all be included, and amongst older year groups there will be absences);
2. It will prove feasible to track the responses from the same pupils over time. The assumption is that for around 50% of the surveys at follow-up, we will have matched baseline data;
3. For single year group analysis, the average sample size per school will be 30 completed surveys per timepoint;
4. The intra-class correlation coefficient is assumed to be 0.2. This is a value typical for school based studies [29].

### Blinding

While it is not possible to blind trial participants in schools or programme facilitators delivering the Keeping Safe training to schools, outcome data will be obtained through self-report measures for teachers, parents and Primary three to Primary seven children thus reducing potential outcome assessor bias. Agency volunteers who will be collecting one to one interview data from Primary one and Primary two children will be blinded to school allocation status. Data processors and the trial statistician will also be blinded in this regard.

### Consent

Six Ambassador Schools with previous experience of taking part in an RCT or other sensitive research have been consulted and involved in developing the evaluation materials and approach to consent that will be used in the Keeping Safe RCT. This has led to the development of information and guidance booklets on the evaluation for parents and teachers, as well as video resources for children.

The process of securing valid informed consent from school stakeholders will begin with recruitment information sessions for school leaders and the provision of resources to enable them to engage their whole school staff and parents. This includes an animated project explainer video; project leaflet; project webpages and links to TV and radio media coverage. Full information on the research will be provided including: what’s involved; why it is being carried out; who will be involved; how the data will be stored and used; measures to safeguard welfare of all participants; and ethical procedures. An RCT infographic will be made available to promote understanding of what randomisation and control group assignment means at school level. Further to the school agreeing to take part, a memorandum of understanding will be used to agree processes, expectations and outputs including procedures for dealing with disclosures of abuse and complaints about the research process/team.

Written ‘process informed consent’ will be sought from all parent, teacher and child participants. Written parental consent will be sought for the participation of all children at baseline and all new school participants (e.g. new intake of primary one children) over the two-year period of the evaluation. Once written parental consent has been provided, an opt-out letter will be sent to parents each year at successive data collection time-points. Once parental consent has been secured, the consent of the children themselves will be sought. Children will watch a specifically designed consent video before completing surveys at each time point. This will inform them about the evaluation and the consent process and will provide practical ways that children can manage their ongoing consent or withdraw at any stage. The video also explains the agency’s confidentiality policy to help the children understand that if they disclose information that gives rise to concerns about their safety or that of another child, the agency may need to link with their school safeguarding team to ensure they are safe. Children will be asked to indicate, by ticking a box on the paper survey or clicking a button on the online survey, whether they consent to take part. The information provided in the explainer video will be presented verbally to children in Primary one and Primary two by a trained volunteer from the agency’s schools service. Children in Primary one and Primary two will be asked to provide verbal assent to their participation. Children with mild-moderate special educational needs and disabilities, and children for whom English is an Additional Language, will be provided with additional support as available in the school to support the securing of their process informed consent to take part.

As the evaluation concerns abuse, it may lead some children to recall past or current sensitive experiences. The ongoing voluntary involvement of children known by the school to have experienced abuse or to be subject to ongoing concerns/cases will be agreed on a one to one basis with the Designated Teacher for Child Protection in the school in conjunction with the child/parents as appropriate. This is important to minimise and prevent personal or social harm. Furthermore, teachers will be provided with a guidance booklet as well as professional development training and resources, to guide them on how best to manage engagement of, and data collection from these children. The project team will be available to offer support to schools and parents on these issues. All children will receive a copy of agency resource booklets on completion of data collection. This is to bring home and help them chat through any concerns with their parents. Participants will have access to a report on the results of the trial once it has been concluded.

## Methods

The evaluation will consist of three rounds of data collection: pre-assessment (baseline), post-assessment one (1 year after baseline) and post-assessment two (2 years after baseline). Data will be collected from children using a composite survey comprising existing and adapted standardised measures across the keeping safe concepts of bullying, physical, sexual, emotional and domestic abuse and neglect as well as anxiety. The content and administration of the survey (online and paper versions available) have been tailored to ensure age and developmental appropriateness and several different versions will be used with different age groups.

Children in Primary four through to Primary seven (ages seven to eleven) will complete online versions of the surveys. Children in Primary three (age six) will complete a paper version in line with their psychomotor development. All children’s surveys consist of self-report measures and will be completed under classroom test conditions supported by classroom teachers and learning assistants. ‘A Guide to Evaluation for Teachers’ booklet will be provided to all classroom teachers to standardise the data collection process across all schools and support school staff collect data from children on these sensitive issues in a manner that safeguards the welfare of the children and is child friendly. The youngest children in Primary one and Primary two (ages four to six) will complete their surveys in one-to-one interview-style sessions with trained volunteers from the non-profit agency schools’ service. Additional school-level administrative behavioural outcome data will be collected via school survey children’s disclosure / teacher reports of children’s maltreatment experiences.

All classroom teachers will be invited to complete a self-report survey with online and paper versions available. Fidelity data on completion of the blended package of training will be collated from the analytics function on the e-learning training programme. Self-reported fidelity to programme implementation in the classroom will be collated using a fidelity monitoring form. Information on school context (size, sector, management type) will also be collected from school administrative data.

All parents will be invited to complete a self-report paper/online survey. Information on parents’/carers’ personal profile (gender) parenting profile (age of children, no of children, relationship to child) home context (Postcode and household income) will be collected via the parent survey.

### Measures

Copies of the outcome measures are available on request from the Principal Investigator.

### Child measures

The children’s surveys will consist of a composite of the measures, outlined below, and a number of versions will be used with different age groups. The children’s measures will be administered at each of the three time points.

#### Demographics

Demographic information will be collected from the children in Primary three through to Primary seven, including gender, age, and primary care giver information.

#### Olweus bullying questionnaire [55]

The two Global Items from the Olweus bullying questionnaire will be used to assess children’s self-reported frequency of bullying perpetration/victimization. These items are culturally appropriate and have been used in the Department of Education survey of bullying in Northern Ireland schools over consecutive years [56]. Olweus’ definition of bullying will be included to promote construct and content validity of these items [31].

#### Children’s knowledge of abuse questionnaire (CKAQ) [57]

The CKAQ is a 33-item self-report questionnaire which will be used to assess children’s (aged six to twelve) knowledge of sexual abuse with a ‘True/False/Not Sure’ format. Responses are scored 0 for incorrect and 1 for correct. Scores will be summed to provide a total sexual abuse ‘knowledge’ score. The CKAQ has strong internal reliability (α = .87) and good test-retest reliability (*r* = .88) [57]. This measure is culturally appropriate and has been used in previous needs assessment research in Northern Ireland schools [42].

#### Revised Children’s manifest anxiety scale 2nd edition (RCMAS-2) [58]

The RCMAS-2 Short Form consists of ten yes/no items and will be used to determine whether programme participation has any negative impact on children’s anxiety levels. A recent US study reported good internal reliabilities in a sample of children aged seven to twelve (α = .78) [59].

#### What if situations test [60]

The What-If Situations Test (WIST) is a six-item scenario-based measure designed to assess six prevention skills: two recognition skills and four personal safety skills. Specifically, they assess children’s (age five to twelve) ability to recognise the appropriateness/inappropriateness of the situation and their ability to refuse, escape, tell and report. The WIST includes a practice scenario, three ‘appropriate request’ scenarios and three ‘inappropriate request’ scenarios. Five additional ‘inappropriate request’ scenarios were added to this measure to include non-contact, online, and peer-to-peer abuse scenarios. Local stakeholders, safeguarding experts and children were engaged in the process of developing the new scenarios. Short seven to ten second animations were developed to accompany each of the 12 scenarios. These will be played via the online survey or on the interactive whiteboard in the classroom for the children in Primary three through to Primary seven (ages six to twelve). Stills from the animations will be used with the children in Primary one and Primary two (ages four to six). Recognition skills are scored 0 for incorrect and 1 for correct. Personal safety skills (inappropriate requests only) are scored from 0 to 2, depending on effectiveness of child’s response. Scores will be summed to provide a total score for each personal safety skill and a WIST total skill score. The original WIST measure has good-strong internal reliabilities (α = .75 to .90) and good test-retest reliabilities (*r*=. 71 to .84) [61].

#### Child-teen witness to woman abuse questionnaire [62]

The ‘Knowledge/Attitudes to Woman Abuse’ sub-scale of the ‘Child/Teen Witness to Woman Abuse questionnaire’ is a 10-item sub-scale that will be used to assess children’s knowledge of domestic abuse using a ‘True/False/Not Sure’ format. This measure is culturally appropriate and has been used in previous needs assessment research in Northern Ireland schools [42]. Responses are scored 0 for incorrect and 1 for correct. ‘Not Sure’ responses will be scored as incorrect. Scores will be summed to provide a total domestic abuse knowledge score.

#### The multidimensional neglectful behaviour scale [63]

Ten items from the original 38-item Multidimensional Neglectful Behaviour Scale will be used to assess children’s knowledge of potentially neglectful scenarios. The items will be adapted and presented as statements (i.e. *It is neglect when…*). Children will respond by answering true, false or not sure. Responses are scored 0 for incorrect and 1 for correct. Scores will be summed to provide a total neglect knowledge score.

#### Self-efficacy sub-scale [64]

The ‘Self-Efficacy Sub-scale’ is a 5-item sub-scale that will be used to assess children’s perceived self-efficacy to keep safe in abusive situations using a ‘1- Really sure I can do it/ 2- I might be able to do it/ 3- Not sure I can do it’ format. An additional 4 items developed through consultation with local stakeholders and safeguarding experts were added to this measure to include situations involving online abuse, cyberbullying, and neglect. Moderate internal reliability (α = .56) was reported for the original measure [64].

An additional response option - ‘*I don’t want to answer this*’ – will also be included for every question to provide children with option of opting out of answering questions that they might find sensitive.

### Parent measure

The parent survey is a composite of the measures outlined below. Parents will be invited to complete this measure at each of the three time points.

#### Demographics

Five items will be used to collect parent and family demographics, including parent/carer gender, relationship to child, number and age of children and home postcode.

#### Parenting and child sexuality questionnaire [65]

Two scales from the Parenting and Child Sexuality Questionnaire [65] were adapted to include topics specific to the Keeping Safe programme. Six items will be used to assess parent’s knowledge of sensitive keeping safe issues; 11 items will be used to assess parents’ confidence to talk to their children about keeping safe. Parents will respond on a 4-point Likert scale from ‘strongly agree’ to ‘strongly disagree’ for knowledge and a 5-point Likert scale from ‘certain I can’ to ‘certain I can’t’ for confidence. Scores will be summed to provide total ‘knowledge’ and total ‘confidence’ scores. Good-strong internal reliabilities were reported for the original knowledge and confidence scales (α = .77 and .95 respectively) [65]. A further four items will be used to assess parents’ current and future confidence to talk to their children about keeping safe [66].

### Teacher measure

The teacher survey is a composite of the measures outlined below. Teachers will be invited to complete this measure at each of the three time points.

#### The teacher willingness to teach sexual health education questionnaire [67]

The teacher comfort subscale will be used to assess teachers’ knowledge and comfort levels in relation to teaching sensitive keeping safe messages. It has been adapted to include topics specific to the Keeping Safe programme. Teachers will respond on a 5 point Likert scale from ‘extremely comfortable’ to ‘not at all comfortable’ for comfort and ‘extremely knowledgeable’ to ‘not at all knowledgeable’ for knowledge. Scores will be summed to provide a total ‘comfort’ score and total ‘knowledge’ score.

#### The survey on perceived confidence in, and attitudes towards, approaches to teaching and learning about sensitive issues in Health & Personal Development [68]

Twelve items from part 2 of the survey will be used to assess teachers’ confidence in managing sensitive issues. This measure has been adapted to focus on the classroom management issues that pertain to the Keeping Safe programme. Teachers will respond on a 5 point Likert scale from ‘strongly agree’ to ‘strongly disagree’. Scores will be summed to provide a total ‘confidence’ score.

#### School-level environment questionnaire – South Africa (SLEQ-SA) [69]

Thirty-five items across six sub-scales will be used to assess teachers’ perceptions of their school culture. Teachers will indicate on a 5 point Likert scale from ‘never’ to ‘always’ how often different practices take place in their school. Internal consistencies for the sub-scales range from α = .69 to α = .92.

### Retention

The evaluation team will endeavour to maximise participant retention throughout the course of the trial. Schools will be provided with resources including templates of school development plans and programme calendars to demonstrate how they can incorporate the programme and evaluation into their own school development planning processes. Schools will also be supported to engage and secure parental support through information sessions and parent evenings. Technical support will be provided to promote programme implementation in intervention schools. Advice and support will also be provided to assist schools with the completion of the teachers’ and children’s surveys in school. Opportunities to seek additional information and support will be available to teachers and parents to promote their retention within the trial and completion of evaluation surveys. Reminder opt-out consent letters will be issued to parents at the beginning of each round of data collection to ensure they remain fully informed and engaged throughout. Regular communications via e-mail and newsletters will be used to update schools on the trial and acknowledge their ongoing support. While every effort will be made to promote school engagement and retention throughout the trial, participants may withdraw at any time for any reason. It will not be possible to carry out follow-up assessments with any participants who have withdrawn from the study.

### Confidentiality

All outcome data will be identified by a unique ID only to ensure participant confidentiality. The unique IDs will also be used to enable matching of data across the timepoints of the trial. An electronic file containing participant names and unique IDs will be stored on a secure network to which only the evaluation team will have access. No identifying information will be recorded on completed data forms. All data collected will be stored anonymously with categorical labels being used to identify according to stakeholder group, school type, school sector, age of pupil, rural urban school. Categorical variables will be held separately to ensure that the statistician is unable to identify individual cases or responses within the data set.

In accordance with agency ethics guidelines, all completed forms (online and paper) will be reviewed for child protection concerns. If a concern or disclosure is identified, the concern, along with the unique ID will be passed to the Programme Development and Implementation Lead who will decide the action required. If necessary, the unique ID can be used to identify the child to whom the concern relates in order to facilitate contact with the child’s school and ensure appropriate safeguarding procedures are put in place. All participants will be made aware of this prior to the start of the evaluation. Any documentation containing identifying personal information, including consent forms will be stored in a locked cabinet to which only the evaluation team will have access. Results from the trial will be reported in a way that ensures no individual school, teacher, parent or child is identifiable.

### Data management

Original survey forms will be returned to the agency at the end of each round of data collection. A secure courier service that has been assessed to satisfy agency procurement policies will be used to transport the data from schools. Original survey forms will be entered onto SPSS by trained blinded research support staff and subsequently stored in an off-site secure storage facility. During the data entry process all files will be kept in locked office cabinets. Regular checks will be carried out for quality control purposes including range checks, valid values and spot checks against original survey forms. Any modifications to data entered will be recorded in a data entry log. There will be significant investment in establishing and maintaining strong relationships and open channels of communication with school level stakeholders including teachers and parents to ensure that data is collected with process informed consent and within a context that safeguards the welfare of children and prevents harm. A queries log will be used to document and track all concerns or queries raised, and capture learning for data collection across the trial duration. A data monitoring committee will not be required for this trial. The conduct of this trial, including modifications to the protocol (trial registry record to be updated annually), will be monitored via quarterly reporting and meetings with the Department of Education and audit by the Department of Finance and Personnel of the Northern Ireland Executive.

Online data storage will be managed by Snap Surveys Ltd. who are independently audited and certified by Bureau Veritas as being compliant with ISO 27001, the internationally recognised gold standard for information security systems. Snap’s UK and US based data centres are hosted at Rackspace, an ISO 27001 certified organisation running SAS 70 / SSAE 16 certified data centres.

### Planned analyses

The data structure within the study is hierarchical, i.e., occasions are nested within the children at level-1 (occasions and individuals are at the same level because a multivariate approach will be taken to the analysis, where this is practical). Each child is nested within a class (level-2), and these classes are nested within the schools (level-3). Variables appropriate to each level will be used in the analysis. At level-1 change in scores across the stages of assessment will be examined within the context of individual variation within and between individuals and the possible factors that might influence these differences, e.g., level of understanding, social background etc.; at level-2 (classroom) the fidelity to the programme will be examined in the context of its effect on the results.

Data will be initially summarised using frequencies, measures of central tendency and spread. The different constructs (examples) will be analysed in two different ways. Equally weighted summed scores will be used where constructs can be empirically shown using measurement models that can be described as belonging to one dimension. In this event the analysis will proceed within the linear mixed models option in SPSS. This will allow both random and fixed effects to be examined in the context of longitudinal data with missing data handled under the assumption of missing at random [70–73].

A second more rigorous approach will also be taken to the analysis [74]. In this option, a structural equation modelling (SEM) approach will be taken, where the constructs will be evaluated across the three points in time, using tests of factorial invariance. A multigroup approach, within the SEM analysis, will be used to test differences across the conditions.

Clustering at the various levels will be considered through the application of multilevel models, as described above, and in some cases using a sandwich estimator (Huber-White) to take account of the non-independence. In the latter case, an adjustment is made to the standard errors and the chi-square test of model fit. The analyses will be conducted within SPSS, STATA or Mplus. Interim analyses focussed on primary outcomes will be conducted with baseline and time 1 data. The results will be shared with the project funder to inform the Department of Education’s strategic business planning process. Interim results will only be reported to schools once time 2 data collection has ended.

### Access to data

The research team will have access to the final data set for preparing the data (i.e. matching data across time points) for analysis. An independent statistician, external to the agency and project will be commissioned to analyse the final data set and will be blinded to school intervention status.

## Discussion

This trial seeks to rigorously evaluate the effectiveness of the Keeping Safe programme that will be taught to children aged between four and 11 years in 80 primary schools across Northern Ireland. This programme will be evaluated in the first cluster randomised controlled trial of a school-based child maltreatment prevention programme in Northern Ireland, Ireland and the United Kingdom. Keeping Safe is a novel maltreatment prevention programme in that it adopts a ‘whole school’ organisation model with components targeting children, staff, parents and the whole school community. Moreover, it seeks to address all forms of maltreatment and includes content on neglect, as well as sexual abuse carried out online or using digital technology, and perpetrated by other children, emerging areas often not included in other programmes [22, 26, 28].

Randomisation will occur at school-level and outcome data will be collected at multiple time points: baseline (T0), end of year one of programme implementation (T1), and end of year two of programme implementation (T2). We hypothesise that children in the intervention schools (at T2) will demonstrate increased knowledge of key programme concepts as well as increased self-efficacy and skills to keep safe in situations of maltreatment. We are seeking to use a proxy measure of children’s skills (What If Situations Test) [60] and school level data on children’s disclosure of maltreatment to measure behavioural outcomes [35]. We also hypothesise that teachers in intervention schools (at T2) will report increased levels of comfort and confidence when teaching programme concepts while parents of children in these schools will report increased comfort and confidence in talking to their children about programme concepts. Further to this, we hypothesise that there will not be an increase in children’s anxiety (at T2), as a result of taking part in the trial [29, 33].

This RCT will address gaps in existing evidence regarding the effectiveness of school based child maltreatment prevention programmes. In addition to the rigorous experimental study design, this trial seeks to measure behavioural outcomes through the collation of children’s disclosure data captured within the child protection system in Northern Ireland [35]. Moreover, this trial will increase our understanding of consenting and collecting sensitive data from young children, adapting and using standardised measures with children of various ages and the administration of a complex trial in a busy school environment. Keeping Safe is a comprehensive multi-component whole-school maltreatment prevention programme to be delivered within a public health framework. This trial is important to determine programme effectiveness and wider use in schools across Northern Ireland and the United Kingdom.
